# Accounting for the Structure–Property Relationship of Hollow-Fiber Membranes in Modeling Hemodialyzer Clearance

**DOI:** 10.3390/polym16243491

**Published:** 2024-12-14

**Authors:** Anton Kozmai, Mikhail Poroznyy, Violetta Gil, Dmitrii Butylskii, Dmitry Lopatin, Aleksey Rodichenko, Igor Voroshilov, Artem Mareev, Victor Nikonenko

**Affiliations:** 1 Membrane Institute, Kuban State University, 149, Stavropolskaya Str., 350040 Krasnodar, Russia; porozhnyj@mail.ru (M.P.); violetta_gil@mail.ru (V.G.); d_butylskii@bk.ru (D.B.); a.a.mareev@bk.ru (A.M.); v_nikonenko@mail.ru (V.N.); 2 LLC “NSK”, 353204 Dinskaya, Russia; lopatin_d@nskomp.ru (D.L.); rodichenko_a@nskomp.ru (A.R.); 3 LLC “KKZ”, 353204 Dinskaya, Russia; gendir@kkzav.ru

**Keywords:** hemodialysis, membrane, structure–property relationship, clearance, mathematical modeling, urea, creatinine, phosphate, porosity

## Abstract

The relevance of the hemodialysis procedure is increasing worldwide due to the growing number of patients suffering from chronic kidney disease. Taking into account the structure of dialysis polymer membranes is an important aspect in their development to achieve the required performance of hemodialyzers. We propose a new mathematical model of mass transfer that allows hollow-fiber membrane structural parameters to be taken into account in simulating the clearance (*CL*) of hemodialyzers in a way that does not require difficult to achieve close approximation to the exact geometry of the membrane porous structure. The model was verified by a comparison of calculations with experimental data on CL obtained using a lab-made dialyzer as well as commercially available ones. The simulations by the model show the non-trivial behavior of the dialyzer clearance as a function of membrane porosity ($f_{p}$) and the arrangement of pores (α). The analysis of this behavior allows one to consider two strategies for increasing the CL of the dialyzer by optimizing the polymer membrane structure: (1) creating a membrane with a well-structured pore system (where α → 1) since doubling α at a high enough $f_{p}$ can lead to an almost tenfold increase in CL; (2) increasing the porosity of the membrane characterized by a random arrangement of pores (α → 0), where, at a relatively low α, a sharp increase in CL is observed with a small increase in $f_{p}$ over a certain threshold value.

## 1. Introduction

An analysis of the hemodialyzer and dialysis filter market reveals that it will reach USD 132.5 billion by 2030, with a projected compound annual growth rate of 6.7% [1]. Such market dynamics are conditioned by the increase in the number of people in need of renal replacement therapy (RRT), which is primarily due to the growth in the elderly population that suffers from chronic kidney disease (CKD) in significantly greater numbers than other age groups [2]. According to a report on CKD prevalence in the United States (2023) presented by the Centers for Disease Control and Prevention, 34% of people aged 65 years and older suffer from CKD, followed by 12% of people aged 45 to 64 years and 6% of people aged 18 to 44 years [3]. However, according to the recent “World Population Prospects” report by the United Nations [4,5], the population aged 65 and older is growing faster than other age categories. In 2020, the percentage of this age group in the general population was 10%; however, by 2050, it is expected to increase to 16%, and by the 2080s, it is predicted to exceed 20%. An important factor in the prevalence of CKD is that CKD is a common complication of pathologies such as diabetes mellitus and arterial hypertension, which are more common in older people [6,7]. Such statistics may indicate that, in the future, the need for medical services for patients with CKD will increase.

In broad terms, kidney disease is characterized by the loss of the bodys ability to maintain its electrolyte balance and remove metabolic waste products. In acute renal failure, the rapid deterioration of renal function is potentially reversible with proper treatment, but the chronic course of the disease results in the progressive and irreversible loss of this function [8,9]. RRT is mandatory in end-stage CKD. Kidney transplantation may be required to treat CKD; however, it is not able to cover all the patients in need. In addition to the obvious limitation of donor resources, it should be noted that a significant number of patients with end-stage CKD cannot be subjected to kidney transplantation for various medical reasons [10,11]. Thus, in end-stage CKD, patients have to rely on extrarenal blood purification methods, such as hemodialysis and hemofiltration, as well as peritoneal dialysis. Compared to hemodialysis, hemofiltration is a more resource-intensive and technically sophisticated method. However, a number of studies [12,13] do not note significant advantages of one method over another regarding the effectiveness of therapy. Despite a considerable number of arguments in favor of peritoneal dialysis, including a better quality of life [14,15], the vast majority of people worldwide receive RRT in the form of hemodialysis [16]. Moreover, due to the functional deterioration of the peritoneal membrane, patients must switch to hemodialysis after a median of 3.7 years of peritoneal dialysis [17].

A significant trend in the global hemodialysis technology market toward engineering developments designed to improve the efficiency and effectiveness of hemodialysis procedures has been observed. In particular, attention is being paid to improving hemodialysis filters, namely in terms of membrane selectivity/permeability to metabolic waste products [18], hemocompatibility and resistance to biofouling [19,20], as well as adaptation of the filter configuration for specific tasks [21,22]. In addition to the above topics, special attention is paid to a personalized approach to ensure effective treatment since patients with end-stage CKD may have different clinical presentations that do not allow a universal treatment. Taking these aspects into account when improving filters can be key to improving patient outcomes, speeding up dialysis treatment, reducing treatment time and increasing overall patient satisfaction and comfort.

Mathematical modeling (MM) can serve as an important tool for the evaluation, optimization and control of hemodialysis therapy, both for everyday clinical use and for the study of its fundamental principles. Indeed, theoretical works in the field of modeling the hemodialysis process have made a significant contribution to the development of hemodialysis technology. The use of MM has made it possible to achieve certain successes in the field of hemodialyzer design and has also laid the basis for evaluating the results of the hemodialysis process using mass transfer kinetics concepts [23]. The theory of mass transfer in a hemodialyzer was proposed in 1981 [24] and has been applied in many studies [25,26,27]. This theory works by satisfactorily applying it to the transport of low molecular weight substances. For larger molecules, a more complex theory was developed taking into account the diffusion boundary layers adjacent to the membrane [28]. In recent years, with the widespread deployment of high-level programming software, there has been significant progress in the theoretical description of the hemodialysis process. In general, a comprehensive review of current research on the modeling of the hemodialysis process for the study and optimization of dialysis therapy can be found in recent works by Pstras et al. [23] and Miyasaka et al. [29]. Works on MM, describing the kinetics of mass transfer through the membrane, have made it possible to obtain the main parameter characterizing the efficiency of the hemodialysis process, clearance [23,29], which is used, among other things, to compare the cleaning capacity of hemodialyzers.

It should be noted that some existing models use a phenomenological approach to describe the transfer of solutes through the membrane and do not take into account its structural characteristics, which directly determine its physicochemical properties. However, there is another approach focused on the structural parameters of the membrane when determining its permeability. In this approach, the well-established theory of free volume has been long and successfully applied to describe transport across membrane structures [30,31,32]. This theory attributes the main role in determining the transport characteristics of membranes to the structural parameter of tortuosity. Generally, the tortuosity of porous media is defined as a function of the volume fraction of pores (porosity). However, many studies provide rather different mathematical expressions for this relationship [33,34,35,36,37]. This can be explained by the rather vague concept of tortuosity, which has multiple definitions, and, possibly, by the fact that separate scientific groups use geometrical and different physical tortuosities interchangeably [38,39]. In most cases of tortuosity determination via porosity, one drawback is the necessity to assume and set geometrical parameters (the shape and characteristic sizes) of pores. Thus, the accuracy of a model depends on how adequately and closely these hypothesized parameters approximate to the complex structure of porous media.

The aim of this study is to develop a new mathematical model of solute transport in dialysis that takes into account the membrane structure–property relationship and allows for the calculation of clearance and other hemodialyzer parameters. Considering the characterization of the membrane structure, in order to determine tortuosity as a function of porosity, we introduce the modification of the microheterogeneous model, taking into account the presence of only one conducting phase in a membrane, which leads to a relatively simple mathematical apparatus. Within the framework of the model, using porosity (the volume fraction of pores) and the introduced structural parameter of pore arrangement makes knowledge of the phase element shape redundant and removes the restrictions of its a priori assumption, unlike in the abovementioned existing approaches. The model was verified using experimental data obtained with a specially designed hemodialyzer during urea, creatinine and phosphate dialysis. The adequacy of the model was confirmed by a comparison with the clearance simulation of several commercially available hemodialyzers with data provided by their manufacturers.

## 2. Theoretical

### 2.1. Model Formulation

The most common design of hemodialyzers includes a cylindrical casing in which a bundle of hollow-fiber membranes is sealed. Blood is pumped through the fibers, and the dialysate solution is pumped through the compartment formed by the outer surface of the membranes and the walls of the dialyzer casing. Generally, in such a system, three layers where the transport of substances occurs can be distinguished: a membrane and two films of solution adjacent to both surfaces of the membrane. Accordingly, in this paper, the theoretical description of mass transfer through membranes in such a dialyzer is based on the following representation of the membrane system.

**Figure 1:**
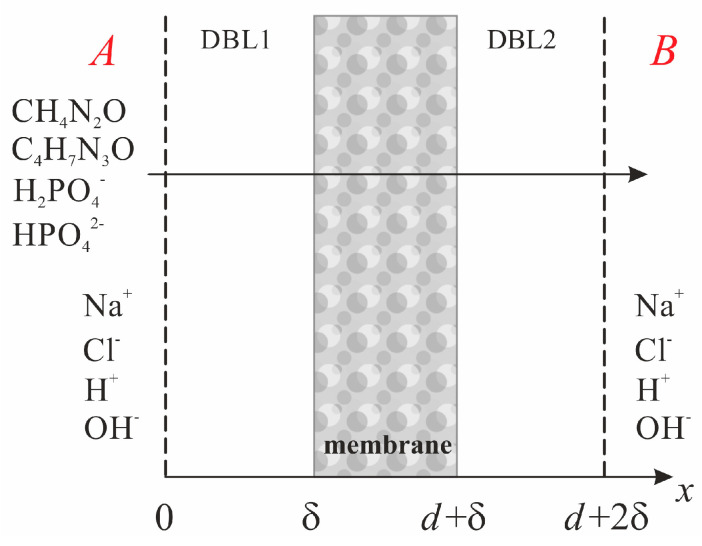
Scheme of the simulated system geometry. DBL1 and DBL2 are diffusion boundary layers adjacent to the membrane at the side of compartments A and B, respectively. The arrow indicates the direction of solutes transport.

The dialysis system under study consists of two compartments: the diluate compartment (*A*) and the dialysate compartment (*B*), which are separated by a membrane (Figure 1).

Along with the components of the hydraulic system (tubing, intermediate tanks), compartments A and B have volumes $V^{A}$ and $V^{B}$, respectively. A model mixed solution containing typical uremic toxins (urea, creatinine and phosphates) and sodium chloride at a given pH circulates through compartment A; an individual solution of sodium chloride at a given pH circulates through compartment B as well as through the intermediate tanks. Phosphoric acid was chosen to simulate phosphate transport.

For the sake of simplicity, it is assumed that the concentration of solutes remains nearly constant along the length of the compartments, i.e., the concentration at any moment in time is the same in the volumes of the compartments and in the corresponding intermediate tanks.

The equilibrium between $H^{+}$ and $OH^{-}$ ions and the water molecules in the solution is described by the following equation:

(1) $$K_{w}=\left[H^{+}\right]\left[OH^{-}\right]=10^{-14}\;{\mathrm{mol}}^{2}\;L^{-2}$$

**Figure 2:**
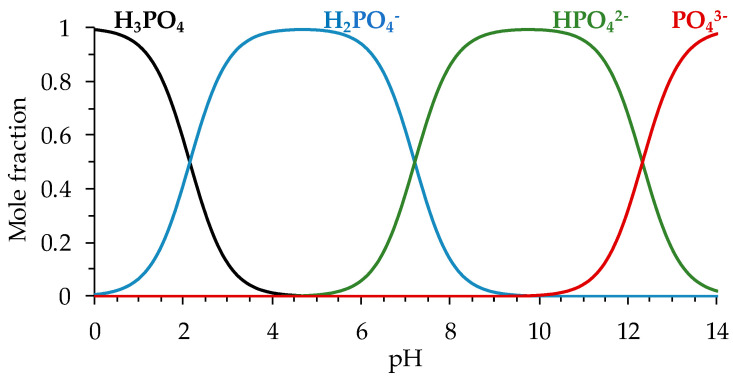
Distribution of the phosphoric acid species (in mole fractions) as a function of pH.

The reactions of urea protolysis are not taken into account in the model since the pH of the solution in the compartments of the system and in the membrane is taken to be equal to 7.4 (the average pH of human blood). At this pH value, the urea in the solution has the form of a neutrally charged molecule (p*K*_a_ of urea is 0.1 [40]). However, the phosphoric acid at this pH exists in the form of singly ($H_{2}PO_{4}^{-}$) and doubly ($HPO_{4}^{2-}$) charged anions (Figure 2). The equilibrium constant of the protolysis reaction

(2) $$H_{2}PO_{4}^{-}+H_{2}O⇄HPO_{4}^{2-}+H_{3}O^{+}$$

at 37 °C is [41]

(3) $$K=\frac{\left[HPO_{4}^{2-}\right]\left[H_{3}O^{+}\right]}{\left[H_{2}PO_{4}^{-}\right]}=6.55\;\times \;10^{-8}\;\mathrm{mol}\;L^{-1}\;(pK\;=\;7.184)$$

Hereafter, the anions $H_{2}PO_{4}^{-}$ and $HPO_{4}^{2-}$ are denoted as $P^{-}$ and $P^{2-}$, respectively; the particles $CH_{4}N_{2}O$ and $C_{4}H_{7}N_{3}O$ are denoted as Ur and Crn, respectively.

The one-dimensional transport of solutes (Figure 1) in the membrane and in the diffusion boundary layers (DBLs) is described by the following set of equations:

– the Nernst–Planck equation

(4) $$J_{i}=-\beta D_{i}\left(\frac{\partial C_{i}}{\partial x}+z_{i}C_{i}\frac{F}{RT}\frac{\partial \varphi }{\partial x}\right)$$

– the electroneutrality condition

(5) $$\sum z_{i}C_{i}=0$$

– the condition of zero current flow

(6) $$\sum z_{i}J_{i}=0$$

– the equation of material balance

(7) $$\frac{\partial C_{i}}{\partial t}=-\frac{\partial J_{i}}{\partial x}$$

where $J_{i}$ is the flux density, $D_{i}$ is the diffusion coefficient, $z_{i}$ is the charge and $C_{i}$ is the concentration of solute i (i = Ur, Crn, $P^{-}$, $P^{2-}$, $Na^{+}$, $Cl^{-}$, $H^{+}$, $OH^{-}$); t is the time; x is the coordinate normal to the membrane surface; β = 1 for the solution and β = ξ for the membrane; ξ is the tortuosity of the conduction pathways in the membrane (see Section 2.3.1); R is the universal gas constant; T is the absolute temperature; *F* is the Faraday constant.

The local change in the phosphoric acid species concentration as a function of pH is expressed using Equation (3), as follows:

(8) $$C_{P^{-}}=\frac{C_{Ptot}}{1+K/C_{H}}$$

(9) $$C_{P^{2-}}=\frac{C_{Ptot}}{1+C_{H}/K}$$

where $C_{Ptot}=C_{P^{-}}+C_{P^{2-}}$ is the total concentration of all phosphoric acid species.

The boundary conditions imply the flux continuity condition at the membrane/solution interfaces

(10) $${\left.J_{i}\right|}_{x=\delta }={\left.{\bar{J}}_{i}\right|}_{x=\delta },\;{\left.J_{i}\right|}_{x=d+\delta }={\left.{\bar{J}}_{i}\right|}_{x=d+\delta }$$

and the concentration continuity condition at the DBLs/solution and membrane/solution boundaries

(11) $${\left.C_{i}\right|}_{x=0}=C_{i}^{A},\;{\left.C_{i}\right|}_{x=d+2\delta }=C_{i}^{B},\;{\left.C_{i}\right|}_{x=\delta }={\left.{\bar{C}}_{i}\right|}_{x=\delta },\;{\left.C_{i}\right|}_{x=d+\delta }={\left.{\bar{C}}_{i}\right|}_{x=d+\delta }$$

where $C_{i}^{A}$ and $C_{i}^{B}$ are the concentration of solutes i in A and B compartments, respectively; the overbar relates to the membrane.

Changes in the concentration of species in compartments A and B, as well as the local concentration in the DBLs and in the membrane, are calculated from the system of equations formed by Equation (7) taking into account Equation (2):

(12) $$\frac{\partial C_{H}}{\partial t}=-\frac{\partial J_{H}}{\partial x}+k_{1}C_{P^{-}}-k_{-1}C_{P^{2-}}C_{H}+k_{d}C_{H_{2}O}-k_{r}C_{H}C_{OH}$$

(13) $$\frac{\partial C_{OH}}{\partial t}=-\frac{\partial J_{OH}}{\partial x}+k_{d}C_{H_{2}O}-k_{r}C_{H}C_{OH}$$

(14) $$\frac{\partial C_{P^{-}}}{\partial t}=-\frac{\partial J_{P^{-}}}{\partial x}-k_{1}C_{P^{-}}+k_{-1}C_{P^{2-}}C_{H}$$

(15) $$\frac{\partial C_{P^{2-}}}{\partial t}=-\frac{\partial J_{P^{2-}}}{\partial x}+k_{1}C_{P^{-}}-k_{-1}C_{P^{2-}}C_{H}$$

(16) $$\frac{\partial C_{Na}}{\partial t}=-\frac{\partial J_{Na}}{\partial x}$$

(17) $$\frac{\partial C_{Cl}}{\partial t}=-\frac{\partial J_{Cl}}{\partial x}$$

(18) $$\frac{\partial C_{Ur}}{\partial t}=-\frac{\partial J_{Ur}}{\partial x}$$

(19) $$\frac{\partial C_{Crn}}{\partial t}=-\frac{\partial J_{Crn}}{\partial x}$$

where $k_{1}$ and $k_{-1}$ are the dissociation and recombination rate constants in the reaction, described by Equation (2); $k_{d}$ and $k_{r}$ are corresponding constants for water.

The numerical solution of the series of Equations (4)–(6) and (12)–(19), taking into account Equations (8) and (9) and the boundary conditions (10) and (11), allows one to find the change in the local concentration of all species in the studied membrane system.

At the beginning of the dialysis process (at t = 0), a uniform distribution of concentrations in the DBLs is assumed, equal to the initial concentrations of the feed solutions. For the sake of simplicity, the concentration of species in the membrane at t = 0 is linearly distributed between its left- and right-hand interfaces.

The numerical solution was implemented in MATLAB (R2014a) software. The membrane, as well as the DBLs, were divided into elementary segments uniformly distributed along the spatial coordinate. For each segment of the system, the algorithm first used by Larchet et al. [42] and described in detail by Mareev et al. [43] was applied. This algorithm involves the use of an explicit Euler finite-difference scheme to approximate the derivatives. The time step was determined in such a way as to satisfy the Courant–Friedrichs–Lewy condition.

### 2.2. Determination of the Dialyzer Clearance

We propose to use the above-described model to calculate the clearance (CL) that a specific dialyzer can provide. The equation for calculating CL reads as follows [29]:

(20) $$CL_{j}=\frac{Q^{A}\left(1-e^{\theta L_{j}}\right)}{1-\frac{Q^{A}}{Q^{B}}e^{\theta L_{j}}}$$

where $\theta =-\pi d_{hf}N_{hf}l_{hf}\left(1-Q^{A}/Q^{B}\right)/Q^{A}$; $Q^{A}$ and $Q^{B}$ are the volumetric flow rates in the diluate and dialysate compartments, respectively ($0\leq Q^{A}<Q^{B}$); $d_{hf}$ and $l_{hf}$ are the inner diameter and the effective length of a hollow-fiber membrane, respectively; $N_{hf}$ is the number of membranes in the dialyzer; $L_{j}$ is the mass transfer coefficient related to solute j (j = Ur, Crn, $H_{3}PO_{4}$).

The value of L is calculated using the following equation:

(21) $$L_{j}=\frac{J_{j}}{\Delta C_{j}}$$

where $J_{j}$ is the flux density of solute j; $\Delta C_{j}=C_{j}^{A}-C_{j}^{B}$ is the difference in concentrations of solute j in compartments A and B, respectively.

Since the non-steady state model (Section 2.1) is applied to calculate $J_{j}$, its value when used in Equation (21) is taken after the onset of a quasi-steady state (about 1 min).

Thus, knowing the value of L, one may calculate the CL for a given set of parameters $Q^{A}$, $Q^{B}$, $d_{hf}$, $l_{hf}$ and $N_{hf}$.

### 2.3. Parameters of the Dialysis Model

The input parameters of the model can be conditionally divided into two main groups: kinetic and structural. There are also several input parameters that characterize the solution and the dialysis system, including the concentration of solutes and the DBL thickness (δ).

The kinetic parameters are the diffusion coefficients of solutes in the membrane (${\bar{D}}_{i}$).

The structural parameters are the volume fraction of pores in the membrane ($f_{p}$) and α, which is the parameter characterizing the arrangement of pores relative to the transport axis.

The output parameters of the model are the local concentration and the local flux density of solutes in the studied dialysis system.

Note that the input parameters were obtained from independent experiments or taken from the literature.

#### 2.3.1. Membrane Structural Parameters

Parameter $f_{p}$ was determined from the mass fraction of water in the membrane (W). The value of W was calculated by the equation

(22) $$W=\frac{m_{sw}-m_{dry}}{m_{sw}}$$

where $m_{sw}$ and $m_{dry}$ are measured values of membrane mass in a swollen and a dry state, respectively.

Thus, knowing the density of a membrane material (ρ), one can calculate the volume fraction of pores as follows:

(23) $$f_{p}=\frac{\rho W}{1+\left(\rho -1\right)W}$$

The structural parameter α was determined using a modified microheterogeneous model by modeling the concentration dependence for diffusion permeability, aligning the theoretical curves with the experimental data obtained for the studied membrane samples in NaCl solution.

The main transport equations of the basic microheterogeneous model can be found in a number of studies [44,45,46]. For a non-charged membrane with a structure formed by two phases (an inert polymer and pores filled with solution), the equation for membrane diffusion permeability (P) obtained from the microheterogeneous model reads as:

(24) $$P=f_{p}^{1/\alpha }D_{s}$$

where $D_{s}$ is the diffusion coefficient of the solution.

Note that the term $f_{p}^{1/\alpha }$ is equivalent to the so-called tortuosity coefficient (ξ). The physical meaning of ξ originates from the fact that the presence of pores randomly distributed within the membrane volume results in the reduced mobility of any solution component. In other words, it takes them longer to cross the membrane.

From the above, it follows that the effective diffusion coefficient of solutes in the membrane is equal to ${\bar{D}}_{i}=\xi D_{i}$ (*i* = Ur, Crn, $P^{-}$, $P^{2-}$, $Na^{+}$, $Cl^{-}$, $H^{+}$, $OH^{-}$). In the first approximation, we assume that the value of ξ is the same for any solute to be transported in the studied membrane system. This assumption is valid because the transported solutes are relatively close in size as well as being rather small compared to the size of the pores in the membrane.

The value of ξ can be determined from the experimental data on membrane diffusion permeability as $\xi =P/D_{s}$.

The use of parameter α in Equation (24) allows one to avoid assumptions about the particular arrangement of pores in the membrane. Equation (24) has a structure similar to that provided by Volfkovich et al. [47], who considered the membrane as a macroporous body. The common peculiarity of approaches based on free volume theory is attributing the difference in the effective diffusion rates of solutes in the membrane to the lengthening of their path associated with the need to go around obstacles.

For a membrane consisting of inert polymers and pores, the value of α is limited by the range 0<α≤1, where α → 0 corresponds to a random arrangement of pores and α = 1 to an arrangement in which the pores are parallel to the transport axis. If the values of $f_{p}$ and ξ are determined, the value of α can be calculated using the following equation:

(25) $$\alpha =\frac{\log f_{p}}{\log\xi }$$

In this regard, α can serve as a criterion for assessing the degree of “ordering” of pores in the membrane.

#### 2.3.2. Diffusion Boundary Layer Thickness

The DBL thickness in the solution adjacent to the membrane surface on the diluate compartment (A) side was estimated using the convective–diffusion model [48]. In the case of the hollow-fiber membrane, which is a long narrow channel, the convective–diffusion model gives an average value of δ equal to 1/3 of the channel width, i.e., 1/3 of the inner diameter of the hollow fiber.

Due to the decisive role of the depleted DBL in mass transfer, as compared to the enriched DBL, we assume that the values of δ at the sides of compartment A and compartment B are the same (Figure 1).

## 3. Experimental

### 3.1. Membrane Manufacturing

Membranes of two geometries were studied: flat and cylindrical (hollow fiber). Three samples of flat membranes were manufactured. For the convenient characterization of the membrane material, flat membranes were studied using a patented dialysis cell according to a well-established membrane characterization method that allows highly accurate data to be obtained [49].

**Figure 3:**
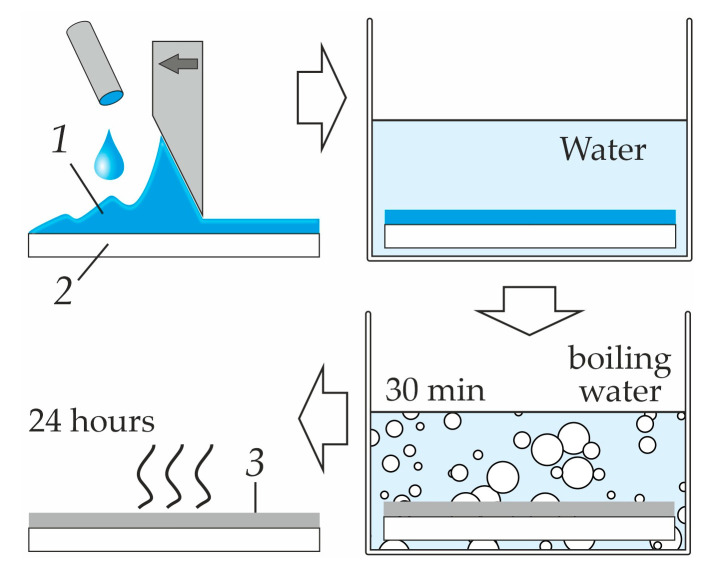
Scheme of the method for preparing flat-sheet membranes. In figure: 1 is the polymer solution, 2 is the glass substrate and 3 is the membrane.

To produce flat-sheet membranes, polysulfone (PSF) PSF-150-V-VD (JSC “G.S. Petrov Institute of Plastics”, Moscow, Russia) dissolved in N-methylpyrrolidone (NMP) (JSC “EKOS-1”, Moscow, Russia) was used. Pore-forming agents capable of dissolving in water were added to the polymer solution: polyethylene glycol (PEG, grade PEG-2000) (JSC “Nizhnekamskneftekhim”, Nizhnekamsk, Russia) or polyvinylpyrrolidone (PVP, grade K30) (Boai NKY Pharmaceuticals Ltd., Jiaozuo, China). To obtain the polymer films of a given thickness, the prepared solution was cast on a glass substrate using the “Doctor Blade” method [50]. The substrate with the cast composition was placed in a container with distilled water to perform phase inversion. The membrane obtained in this way was placed in a container with boiling distilled water for 30 min to remove the residual solvent and dried in air for 24 h (Figure 3).

In terms of strength and structural integrity, the optimal concentration of PSF in the NMP solvent was determined to be 20%. When adding pore-forming agents, the best performance was observed with a content of 30% and 10% of the PSF mass for PEG and PVP, respectively. As a result, three samples of flat polysulfone membranes were obtained (Table 1): a membrane without the use of a pore-forming agent and membranes with the use of PEG and PVP pore-forming agents.

To manufacture a hollow-fiber membrane (denoted as HF-PSF-PVP), a method of dry–wet spinning was applied [51,52]. The polymer solution was obtained using the same PSF and PVP as in the corresponding flat-sheet membrane.

**Figure 4:**
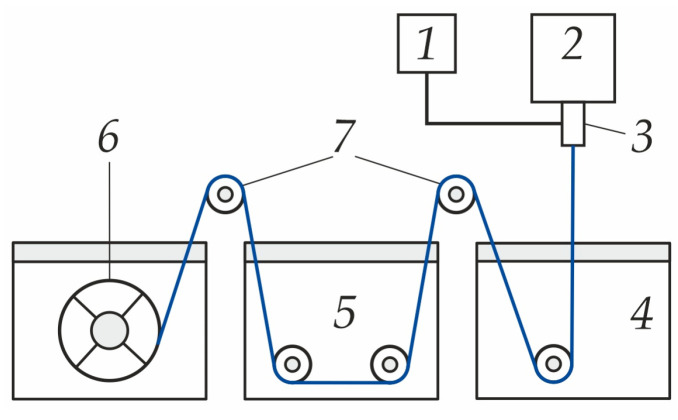
Scheme of the setup for manufacturing a hollow-fiber membrane. In figure: 1 is the polymer feed tank, 2 is the coagulant feed tank, 3 is the spinnerets, 4 is the coagulation bath, 5 is the washing bath, 6 is the winding roller, and 7 is the auxiliary rollers.

The scheme of the setup is presented in Figure 4.

The polymer solution and bore solution are fed from tanks 1 and 2 into the spinneret (3). Passing this way, the polymer solution acquires a tubular shape, forming a hollow fiber. Then, the hollow-fiber membrane enters the coagulation bath (4) and passes through the washing bath (5) via the system of rollers (7) and is wound onto the roller (6).

### 3.2. Membrane Characterization

#### 3.2.1. Diffusion Permeability

The diffusion permeability coefficients for the studied flat membranes were determined in NaCl solutions with a concentration of 0.10, 0.25, 0.50 and 0.75 mol L^−1^ using a two-chamber flow cell according to the method described in detail in [49].

**Figure 5:**
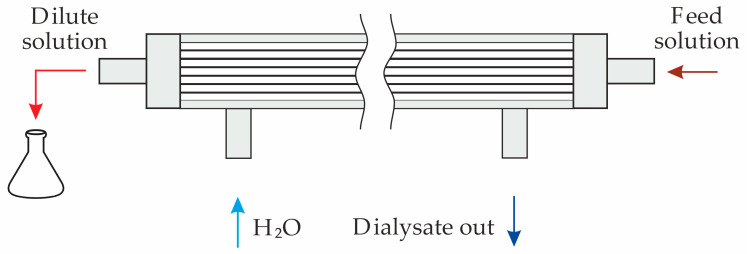
Scheme of the lab-made dialyzer.

The diffusion permeability coefficients for the studied hollow-fiber membranes were determined by a similar method using the same experimental setup, in which the two-chamber cell was replaced by a lab-made dialyzer (Figure 5).

The dialyzer was equipped with 90 hollow-fiber membranes, with the effective length of each fiber set at 15 cm and the diameter at 480 μm. The average hollow-fiber membrane thickness (the membrane “wall”) was 140 μm. The experiments were carried out at a temperature of 25.0 ± 0.5 °C.

#### 3.2.2. Water Content

Before determining the water content, the membrane sample was equilibrated with distilled water. The sample was then removed, and excess water was wiped off the membrane surface with filter paper. To measure the mass of the swollen sample, $m_{sw}$, and the mass of the dry sample, $m_{dry}$, an MB25 moisture content analyzer (Ohaus, Parsippany, NJ, USA) was used. The sample was dried at a temperature of 100 °C until its mass ceased to change. The water content of the membrane (%) was calculated as $WC=100\%\cdot \left(m_{sw}-m_{dry}\right)/m_{sw}$.

#### 3.2.3. Scanning Electron Microscopy

Images of the surface and cross section of the membrane samples in a dry state were obtained using a scanning electron microscope (SEM) (JEOL JSM-7500F JEOL Ltd., Tokyo, Japan). To improve conductivity and enhance image quality, the membrane samples were coated with a thin layer of silver (about 5 nm).

### 3.3. Dialysis

Experiments on the dialysis of individual solutions of urea, creatinine and phosphates were carried out using the lab-made hemodialyzer (Figure 5).

The initial concentrations of urea, creatinine and phosphoric acid in the diluate compartment were taken as equal to 1.67 × 10^−2^, 8.85 × 10^−4^ and 2.9 × 10^−3^ mol L^−1^, respectively. These are typical concentrations in the blood plasma of a 50-year-old patient with CKD undergoing hemodialysis [53,54]. Solutions of a given concentration were pumped through the hollow-fiber membranes (diluate compartment) at a volumetric flow rate of 2 mL min^−1^, while deionized water was pumped through the dialysate compartment of the dialyzer at a volumetric flow rate of 4 mL min^−1^.

In the case of dialysis of the urea solution, a 40 mL sample was taken at the outlet of the diluate compartment. To this sample, 10 mL of n-dimethylaminobenzaldehyde (DMABA) solution (5 g L^−1^) was added for subsequent analysis on a UV-1800 spectrophotometer (TMECO-VIEW, Shanghai Mapada Instruments Co., Ltd., Shanghai, China) at a wavelength of 420 nm, corresponding to the maximum absorption of the urea complex with DMABA, which imparts a yellow–green color to the solution. From the obtained absorption coefficient value, the urea concentration ($C_{Ur}^{out}$) in the sample was determined according to a previously obtained calibration scale for known concentrations.

In the case of dialysis of the creatinine solution, a 5 mL sample was taken at the outlet of the diluate compartment. To this sample, 50 mL of a 1:1 mixture of picric acid (1.75 × 10^−4^ mol L^−1^) and sodium hydroxide (0.3 mol L^−1^) was added for subsequent analysis on a UV-1800 spectrophotometer (TMECO-VIEW, Shanghai Mapada Instruments Co., Ltd., Shanghai, China) at a wavelength of 500 nm, corresponding to the maximum absorption of the creatinine complex with alkaline picrate, which imparts a yellow–red color to the solution. From the obtained absorption coefficient value, the creatinine concentration in the sample was determined using a previously obtained calibration scale for known concentrations.

In the case of dialysis of the phosphate solution, a 1 mL sample was taken at the outlet of the diluate compartment for the subsequent determination of the total phosphate concentration using a DIONEX ICS-3000 (Thermo Fisher Scientific, Waltham, MA, USA) chromatographic system with conductometric detection and a background signal suppression system.

The experiments were carried out at a temperature of 37.0 ± 0.5 °C.

From the obtained concentrations $C_{j}^{out}$ (j = Ur, Crn, $P^{tot}$), the clearance was calculated by the following formula:

(26) $$CL_{j}=Q^{A}\left(1-\frac{C_{j}^{out}}{C_{j}^{in}}\right)$$

where $C_{j}^{in}$ is the feed solution concentration.

## 4. Results and Discussion

### 4.1. Analysis of the Membrane Structural Parameters

**Figure 6:**
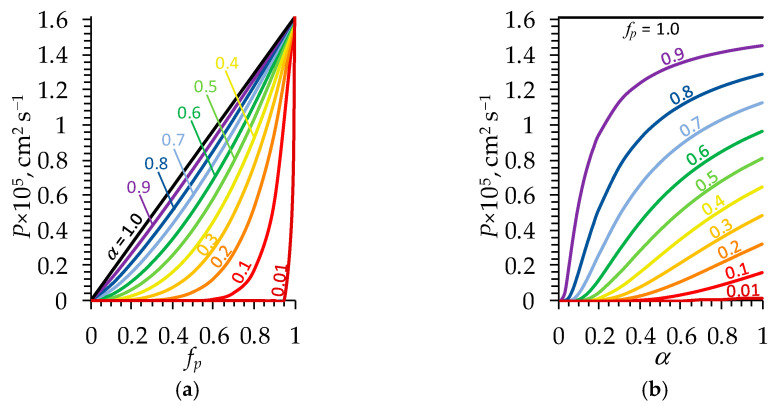
Membrane diffusion permeability coefficient P as a function of (**a**) the volume fraction of pores in the membrane $f_{p}$ at a fixed value of structural parameter α (indicated near the corresponding curve) and (**b**) parameter α at a fixed value of $f_{p}$ (indicated near the corresponding curve). Calculations were made using Equation (24) for the NaCl solution.

Diffusion permeability is one of the most important transport characteristics of dialysis membranes. The effect of the volume fraction of pores $f_{p}$ and structural parameter α on membrane diffusion permeability P was analyzed based on the results of numerical experiments carried out using Equation (24) (Figure 6).

Figure 6a shows that the value of P tends to be the value of solution diffusion coefficient $D_{s}$ with increasing $f_{p}$ at a fixed value of α. Note that in the case of NaCl, $D_{s}=2D_{Na}D_{Cl}/\left(D_{Na}+D_{Cl}\right)$ = 1.61 × 10^−5^ cm^2^ s^−1^ at an infinite dilution at 25 °C. On the other hand, with increasing α at a fixed value of $f_{p}$ (Figure 6b), the value of P tends to be the value $f_{p}D_{s}$ according to Equation (24). It is obvious that the higher the porosity is and the closer the pore arrangement is to a parallel connection, the higher the permeability of the membrane.

As for the pore arrangement, in practice, obtaining membranes with straight pores that pass through is a rather delicate problem. Polyethylene terephthalate track-etched membranes [55] or membranes based on titanium oxide nanotube arrays can serve as an example [56,57]. However, as far as the authors know, these types of membranes are obtained only in the form of flat sheets. This means that a dialyzer with such membranes will have a bulky design. Moreover, despite the fact that titanium has excellent biocompatibility, the abovementioned material is fragile.

If the arrangement of pores in the membrane tends to be random, the dependence of diffusion permeability on porosity is characterized by a steep increase at relatively high values of $f_{p}$ (Figure 6a). From the point of view of the membrane structure, the appearance of a relatively small fraction of additional pores over a certain threshold leads to a significant increase in the variability of conduction pathways and, as a consequence, to an increase in P. Thus, when developing materials for the production of hemodialysis membranes, it is necessary to focus on the increase in their porosity.

In dialysis membrane manufacturing, various pore-forming agents are used to provide the polymeric material with an extensive porous structure. In order to investigate the structure–property relationship of such polymeric material, it is convenient to study this material as flat-sheet membranes because flat geometry allows for simple yet precise characterization using a well-established method [49]. Therefore, to examine the effect of a porous membrane structure on diffusion permeability, three flat-sheet membranes with different porosities attributed to pore-forming agents (Table 1) were studied.

**Figure 7:**
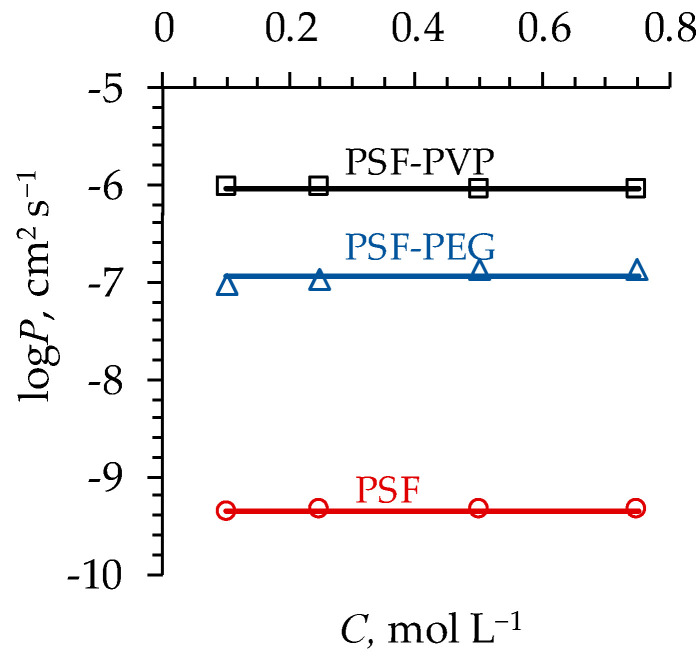
Concentration dependence of the membrane (indicated near the corresponding curve) diffusion permeability coefficient. Markers indicate the experimental data; solid lines indicate the results of calculations with Equation (24).

Experimental and theoretical data on the diffusion permeability of the flat-sheet membranes are presented in Figure 7.

The results of the studied membrane structure characterization carried out using Equations (22)–(24) to determine the best fit between the simulated and experimentally determined diffusion permeability coefficients (Figure 7) are shown in Table 2.

**Figure 8:**
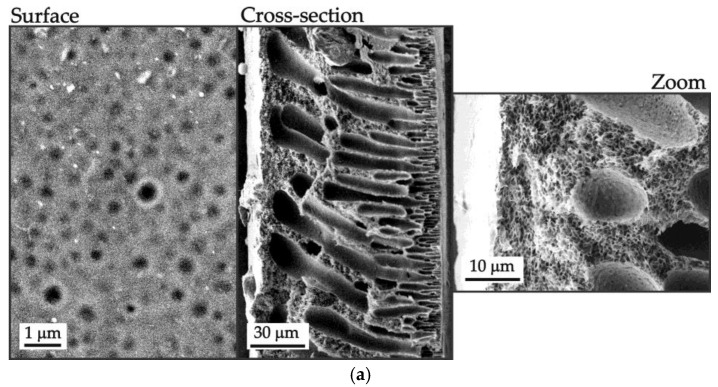

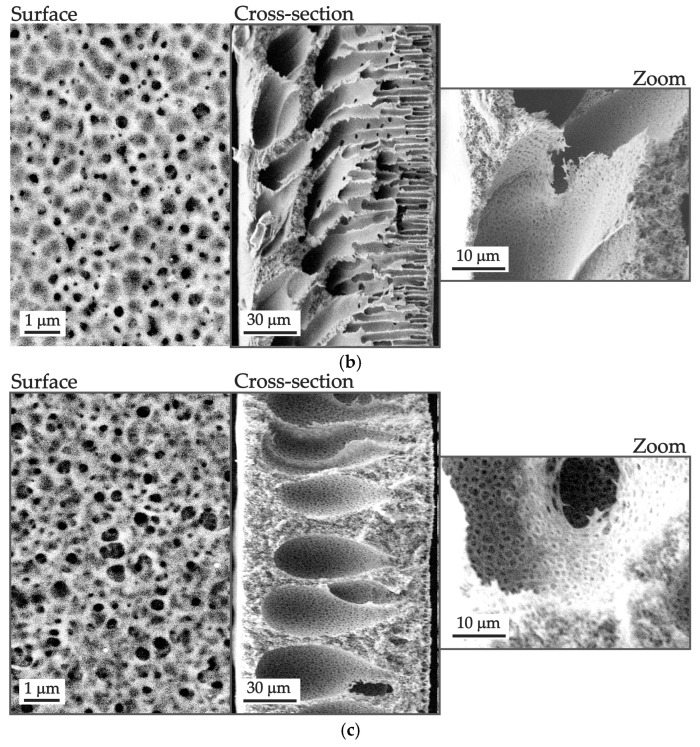
SEM images of surfaces and cross sections (indicated near the figure) of the (**a**) PSF, (**b**) PSF-PEG and (**c**) PSF-PVP membranes.

The PSF membrane, obtained without the use of a pore-forming agent, is characterized by the lowest value of P (Figure 7). A very low diffusion permeability for this membrane can be explained by the low value of α at a relatively low value of $f_{p}$ (Table 2). Pores do not form well-structured continuous domains that can facilitate the transport of solutes between the pore walls (Figure 8a). Low pore density and a small number of open pores on the surface (Figure 8a) also contribute to the low diffusion permeability of this membrane.

As for membranes made with the use of pore-forming agents, their diffusion permeability is two (PSF-PEG) and three (PSF-PVP) orders of magnitude higher than that of the PSF membrane due to greater porosity and a greater pore density on the surface (Figure 8b,c). The pores themselves are larger and, judging by the values of α (and ξ), form continuous conduction pathways along the transport axis better.

Despite the fact that the values of α for the PSF-PEG and PSF-PVP membranes are relatively close to each other (Table 2), the decisive factor in ensuring the relatively high diffusion permeability of the PSF-PVP membrane is the higher value of $f_{p}$ at a low value of α, where the diffusion permeability as a function of $f_{p}$ is characterized by a steep increase (Figure 6a).

The above analysis indicates that PVP should be better than PEG for producing hollow-fiber membranes with a well-developed porous structure. Therefore, a hollow-fiber membrane based on PSF and PVP was manufactured for further investigation (the hollow-fiber membrane manufacturing method is described in Section 3.1).

The characterization of this hollow-fiber membrane (HF-PSF-PVP) yields an effective diffusion permeability coefficient value of 6.9 × 10^−9^ cm^2^ s^−1^. The membrane water content, the volume fraction of pores and the value of the structural parameter determined using Equations (23) and (24) are as follows: W = 0.73, $f_{p}$ = 0.77 and α = 0.083. Note that these values are virtually the same as those determined for the corresponding flat-sheet PSF-PVP membrane: W = 0.74, $f_{p}$ = 0.78 and α = 0.086 (Table 2). The only considerable difference is observed for the values of ξ, which is very sensitive to even the slightest change in parameters $f_{p}$ and α. The decrease in ξ for the hollow-fiber membrane, as compared to the flat-sheet one, reflects the increase in the tortuosity of the pore structure and, as a result, the increase in mass transfer resistance. Consequently, the diffusion permeability coefficient of the HF-PSF-PVP membrane takes a lower value than that of the PSF-PVP membrane (P = 8.9 × 10^−9^ cm^2^ s^−1^).

**Figure 9:**
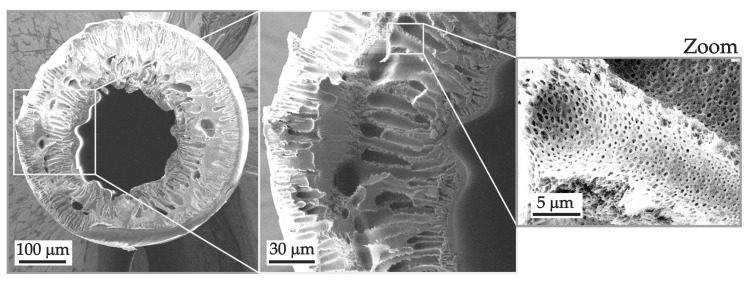
SEM images of the HF-PSF-PVP membrane in cross section.

This may be attributed to the fact that the manufacturing technology has an impact on membrane structure, i.e., on the solvent/non-solvent exchange during the phase inversion fabrication process [52]. In the case of flat-sheet membranes, one side is adjacent to the glass substrate and is not directly exposed to the non-solvent during membrane formation. As a result, large voids in the membrane are formed close to the side opposite the substrate. In the case of hollow-fiber membranes, they are immediately exposed to both the non-solvent bore solution and the water vapor from the spinneret air gap. Therefore, relatively large voids are preferably not formed at any side of the membrane (Figure 9).

A smaller fraction of relatively large voids in the structure of the HF-PSF-PVP membranes compared to that of the PSF-PVP leads to a slightly lower value in their diffusion permeability.

### 4.2. Model Verification

The verification of the proposed model was carried out by a comparison of the experimentally determined and simulated Ur, Crn and phosphate ($P^{tot}$) clearances for the laboratory-scale dialyzer containing HF-PSF-PVP membranes. The parameters of the membranes, their structure (as determined above) and the dialyzer configuration used in the simulations are presented in Table 3.

The initial concentrations of urea, creatinine and phosphoric acid in the diluate compartment were taken as equal to 1.67 × 10^−2^, 8.85 × 10^−4^ and 2.9 × 10^−3^ mol L^−1^, respectively. The effective membrane area for the laboratory dialyzer containing 90 hollow fibers was 80.8 cm^2^.

**Figure 10:**
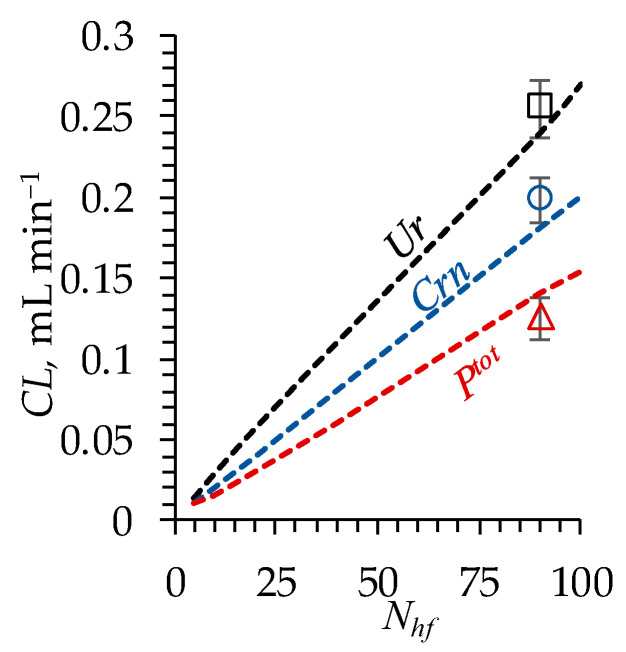
Clearance of urea (Ur), creatinine (Crn) and phosphates ($P^{tot}$) (indicated near the corresponding curve) as a function of the number of hollow fibers $N_{hf}$ in the dialyzer. Markers indicate the experiment; dashed lines indicate the results of simulations.

Figure 10 shows that the experimentally obtained values of the Ur, Crn and $P^{tot}$ clearances are in good qualitative agreement with the results of the simulations.

Therefore, the verification of the model demonstrated its adequacy in describing the clearance of urea, creatinine and phosphate provided by the dialyzer.

### 4.3. Simulation of the Membrane Parameter Effect on Clearance

The question of practical importance when designing a hemodialyzer is which membrane parameters affect CL the most.

The relationship between urea clearance and the hollow-fiber effective length as well as the hollow-fiber inner diameter is analyzed in a review by Miyasaka and Sakai [29]. Below, using the developed model, we analyze the effect of the membrane geometric ($l_{hf}$, $d_{hf}$, d, $N_{hf}$) and structural ($f_{p}$ and α) parameters on the urea clearance of the dialyzer.

The set of parameters presented in Table 3 was taken as a reference. In the simulations, the ratio of volumetric flow rates in the compartments ($Q^{A}/Q^{B}$) was taken to be 3/5.

**Figure 11:**
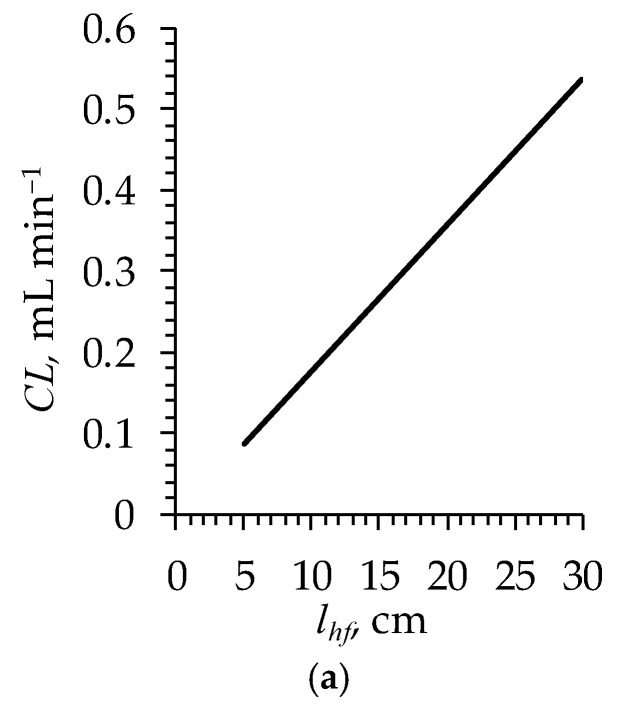

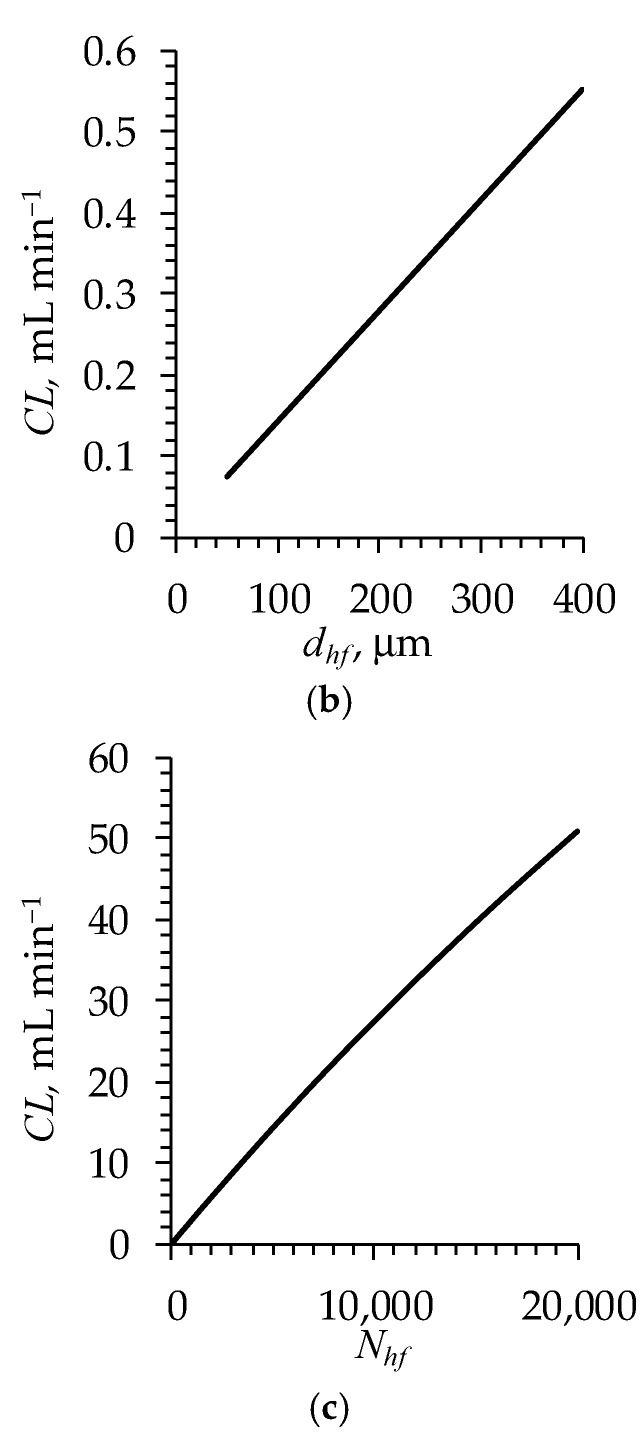
Simulated clearance of urea as a function of (**a**) fiber length ($l_{hf}$); (**b**) fiber inner diameter ($d_{hf}$); (**c**) the number of fibers in the dialyzer ($N_{hf}$). In figures (**a**,**b**) $N_{hf}$ = 90, as indicated in Table 3.

The simulations show the linear (or almost linear) dependence of CL on $l_{hf}$, $d_{hf}$ and $N_{hf}$ (Figure 11).

Miyasaka and Sakai [29] reported the appearance of an extremum in CL vs. $d_{hf}$ and the asymptotic behavior of CL vs. $l_{hf}$ dependencies. Apparently, such nonlinearity is due to the condition that sets a constant value for the effective membrane area ($S_{mb}$), which, in turn, depends simultaneously on a number of membranes and dialyzer configuration parameters. In our simulations, $S_{mb}$ was not constant since we considered the effect of each parameter independently.

**Figure 12:**
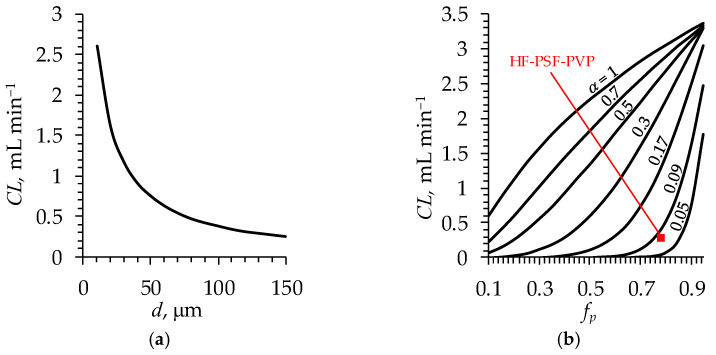
Simulated clearance of urea as a function of (**a**) membrane (fiber wall) thickness (d); (**b**) the volume fraction of pores in the membrane ($f_{p}$) at various values of structural parameter α (indicated near the corresponding curve). The red square marker indicates the experimentally determined value of CL for the lab-made dialyzer containing the HF-PSF-PVP membranes.

A strong increase in CL is observed with decreasing d (Figure 12a), which is explained by the fact that the solute flux is inversely proportional to the membrane thickness (Equation (4)).

Evidently, the higher the porosity is the greater the clearance. However, the shape of the curve (Figure 12b) reflecting this dependence is defined by the value of the structural parameter α: the lower α is the steeper the increase in CL with an increasing $f_{p}$. This allows one to conclude that with relatively high porosity and small values of α, even a small increment in porosity over a certain threshold leads to a significant increase in CL. That is, when designing membranes, special attention should be paid to increasing the porosity. It is also worth looking for ways to create a membrane with a better structured pore system since doubling α at a fixed $f_{p}$ can lead to an almost tenfold increase in CL, as shown by calculations. For example, at a fixed value of $f_{p}$ = 0.8, in the case of α = 0.05, one obtains the value of CL = 0.07 mL min^−1^; however, in the case of α = 0.1, CL = 0.64 mL min^−1^ (Figure 12b).

### 4.4. Obtaining Membrane Structural Parameters by the Analysis of Commercial Dialyzer Clearance

To evaluate the structural parameters of the membranes used in commercial dialyzers, dialyzers from several manufacturers widely represented on the world market were selected: the Nephral ST series from Baxter, the FX series from Fresenius and the Diacap Pro series from B. Braun. According to the manufacturers datasheets [58,59,60], the membrane used in the Baxter Nephral ST series dialyzers has a thickness of 45.5 μm and inner diameter of 210 μm; for the Fresenius FX series dialyzers, the corresponding values are 35 μm and 210 μm; for the B. Braun Diacap Pro series dialyzers, the values are 37 μm and 200 μm. The effective membrane area ($S_{mb}$) and number of fibers ($N_{hf}$) for the studied dialyzers are presented in Table 4.

The difference in the membrane areas is achieved by changing the number of membranes in the dialyzer.

The experimental data (obtained in vitro) on urea, creatinine and phosphate clearance were taken as the volumetric flow rates in the diluate compartment of 200, 300 and 400 mL min^−1^ [58,59,60] if available for the abovementioned dialyzers. The volumetric flow rate in the dialysate compartment was taken as equal to 500 mL min^−1^ [58,59,60].

The model parameters used in the clearance simulations are presented in Table 5. The initial concentrations of urea, creatinine and phosphoric acid were taken as the same, as in the case of the lab-made dialyzer. The number of hollow fibers in the dialyzer was selected in the model so that the calculated membrane area and the effective membrane area provided by the manufacturer were equal to each other. The resulting values of $N_{hf}$ are presented in Table 4.

**Figure 13:**
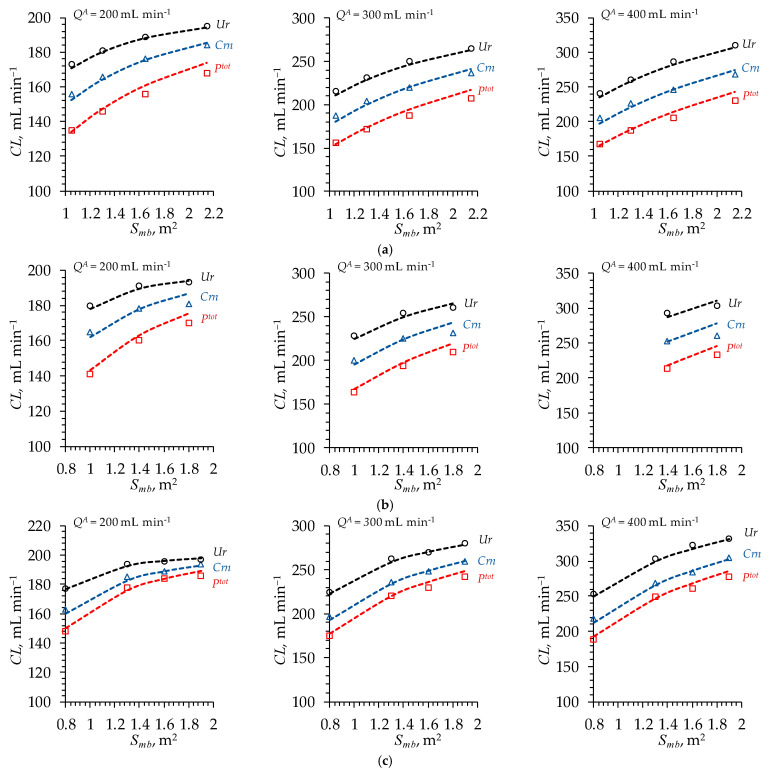
Simulated (dashed lines) and experimentally obtained (markers) (**a**) Baxter; (**b**) Fresenius and (**c**) B. Braun clearance of Ur, Crn and $P^{tot}$ (indicated near the corresponding curve) as a function of the effective membrane area at the volumetric flow rates in the diluate compartment, $Q^{A}$, indicated in the plots.

The parameters listed in Table 5 were used as a single set to simultaneously simulate the CL of all the considered dialyzers from the corresponding manufacturers at all the volumetric flow rates. In all cases, good agreement between the simulated and the experimental CL (Figure 13) was achieved by adjusting only one parameter, ξ (tortuosity coefficient), to the same value: 0.42, 0.45 and 0.8 for the Baxter, Fresenius and B. Braun membranes, respectively.

**Figure 14:**
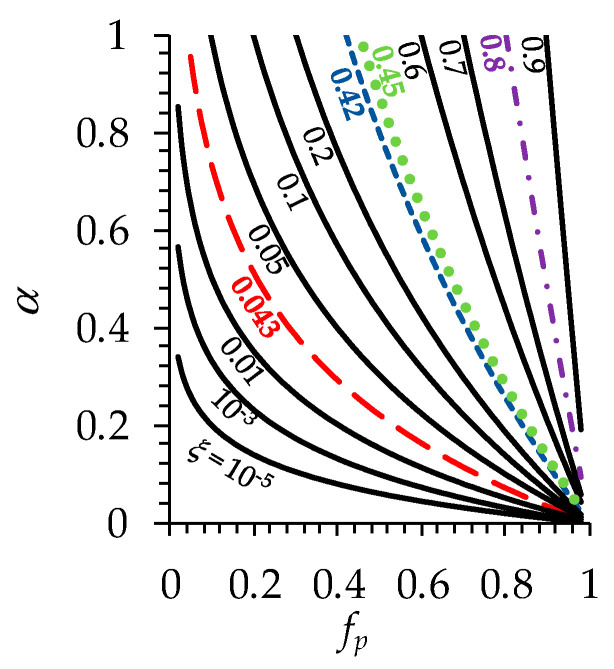
Simulated α vs. $f_{p}$ dependence at various values of ξ (indicated near the corresponding curve). Red-dashed line corresponds to a lab-made HF-PSF-PVP membrane, the blue-dashed line denotes the Baxter membrane, the green-dotted line denotes the Fresenius membrane and the purple-dash–dotted line denotes the B. Braun membrane.

It is worth noting that the value of ξ can be achieved with various combinations of $f_{p}$ and α (ξ = $f_{p}^{1/\alpha }$). As can be seen from Figure 14, with an increase in $f_{p}$ at fixed α and with increasing α at fixed $f_{p}$, the tortuosity coefficient increases (i.e., the tortuosity of the conduction pathways decreases).

Since the values of $f_{p}$ for the commercial membranes are unknown, one can assess the effect of the membrane structure on its performance by analyzing the α vs. $f_{p}$ dependencies obtained for the value of ξ determined for these membranes (Figure 14).

As can be seen from Figure 14, for any value of parameter $f_{p}$, the value of α is considerably higher for the B. Braun membrane than for the other membranes. As for the Baxter and Fresenius membranes, the corresponding curves are rather close to each other.

Let us consider two hypothetical cases: (1) where the volume fractions of the pores in the studied membranes are equal to each other, and (2) where the structural parameter α for these membranes takes the same value.

In the first case, we assign an arbitrary reasonable value, $f_{p}$ = 0.9, to all the commercial membranes. Then, the corresponding values of α are 0.12, 0.13 and 0.47 for the Baxter, Fresenius and B. Braun membranes, respectively. In the second case, where the pore arrangement for all the membranes is the same, for example, α = 0.5, $f_{p}$ takes the values 0.65, 0.67 and 0.89 for the Baxter, Fresenius and B. Braun membranes, respectively. This means that the structure of the membrane from B. Braun is characterized by a more “organized” and developed pore system, i.e., the pore arrangement and their fraction provide less curved conduction pathways, ensuring comparatively high clearance.

**Figure 15:**
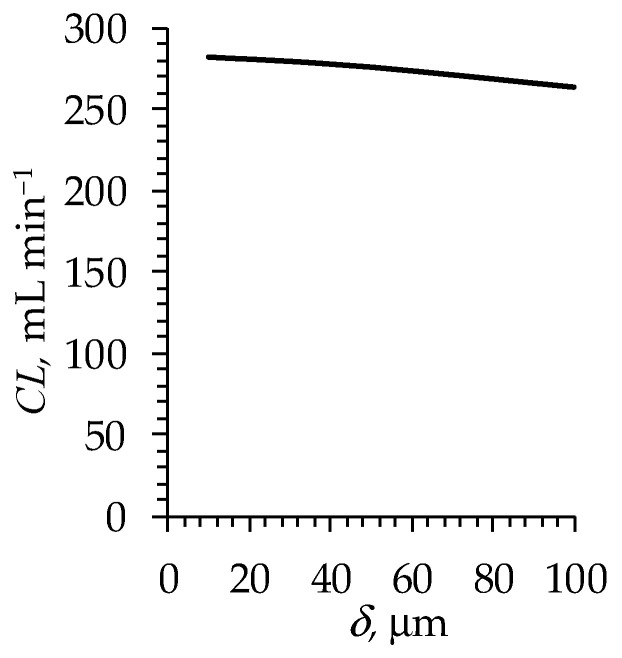
Simulated urea clearance for the Nephral ST 300 dialyzer as a function of the diffusion boundary layer thickness.

Note that the volumetric flow rate should affect the mass transfer rate across the hollow-fiber membranes. The proposed model takes into account the hydrodynamics through the setting of an effective DBL thickness. Despite the simplicity of such an approach, there is good agreement between the experimental and the simulated data on CL at all the considered flow rates. This indicates that the change in $Q^{A}$ (in the considered range of its values 200–400 mL min^−1^) has an effect on δ such that the resulting changes in clearance occur within the error limits of the measurement method (about 10% [58]). This conclusion is supported by the results of the numerical experiments carried out using the model (Figure 15).

As Figure 15 shows, within a relatively wide range of δ values, the value of clearance changes just by a few percent.

## 5. Conclusions

A mathematical model for solute transport through the hemodialysis membrane that takes into account the membrane structure–property relationship is developed. A new approach to determine membrane structural parameters (the volume fraction of pores and their arrangement relative to the transport axis) is proposed. The model is applied to simulate the urea, creatinine and phosphate clearance of (1) a lab-made hemodialyzer containing polysulfone-based hollow-fiber membranes and (2) commercial dialyzers produced by the Baxter, Fresenius and B. Braun companies.

The numerical experiments using the model show the non-trivial behavior of the dialyzer clearance as a function of pore tortuosity in a membrane. This result, together with the fact that the tortuosity of the conduction pathways in the membrane depends on both the porosity and the arrangement of pores, allows one to consider two strategies for increasing the clearance of the dialyzer. The first is to develop methods of manufacturing membranes with a well-structured pore system that may overcome the limitations in CL conditioned by a relatively low volume fraction of pores. The second is to increase the porosity of the membrane to the point where a random arrangement of pores is no longer a substantial hindrance to CL.

Comparison of the results of the clearance simulations with the data provided by the manufacturers of the commercial dialyzers indicates that the proposed model is applicable to the characterization of membranes in dialyzers already available on the market.

Further development of the model involves taking into account the presence of a layer of substances absorbed on the membrane surface and the water flux across the membrane. These modifications will allow for a more accurate description of the transport of high-molecular compounds and the balance of electrolytes, which are the osmotic factors in the dialysis process.

## Figures and Tables

**Table 1:** Flat-sheet membranes under study.

Membrane	Polymer	Pore-Forming Agent
PSF	PSF 20%	-
PSF-PEG		PEG 30%
PSF-PVP		PVP 10%

**Table 2:** Structural parameters of the studied flat-sheet membranes.

Membrane	$f_{p}$	α	ξ	W
PSF	0.60	0.049	2.7 × 10^−5^	0.55
PSF-PEG	0.67	0.081	0.007	0.62
PSF-PVP	0.78	0.086	0.056	0.74

**Table 3:** Model parameters used in the clearance simulations.

Parameter	Value	Dimension
$f_{p}$	0.77	-
α	0.083	-
δ	63	μm
$l_{hf}$	15	cm
$d_{hf}$	190	μm
d	140	μm
$N_{hf}$	90	-

**Table 4:** Effective membrane areas [58,59,60] and the calculated number of hollow fibers for the studied dialyzers.

Baxter	$S_{\mathrm{mb}}$, m^2^	$N_{\mathrm{hf}}$	Fresenius	$S_{\mathrm{mb}}$, m^2^	$N_{\mathrm{hf}}$	B. Braun	$S_{\mathrm{mb}}$, m^2^	$N_{\mathrm{hf}}$
ST 200	1.05	6400	FX 5	1.0	7600	Pro 08H	0.8	6400
ST 300	1.30	7900	FX 8	1.4	10,600	Pro 13H	1.3	10,400
ST 400	1.65	10,000	FX 10	1.8	13,600	Pro 16H	1.6	12,700
ST 500	2.15	13,000				Pro 19H	1.9	15,100

**Table 5:** Model parameters used in the commercial dialyzer clearance simulation.

Dialyzer	δ, μm	$d_{\mathrm{hf}}$, μm	$l_{hf}$, cm	d, μm
Nephral ST	70	210	25	45.5
FX	70	210	20	35.0
Diacap Pro	65	200	20	37.0

## Data Availability

The data presented in this study are available on request from the corresponding author. The data are not publicly available due to privacy.

## Abbreviations

**Designation**	**Description**
Abbreviations	
DBL	Diffusion boundary layer
NMP	N-methylpyrrolidone
PSF	Polysulfone
PVP	Polyvinylpyrrolidone
SEM	Scanning electron microscopy
Symbols	
C	Concentration
*CL*	Clearance
d	Membrane thickness
D	Diffusion coefficient
$d_{hf}$	Inner diameter of the hollow-fiber membrane
F	Faraday constant
$f_{p}$	Volume fraction of pore space in the membrane
J	Flux density
K	Equilibrium chemical reaction constant
$k_{1}$ and $k_{-1}$	Dissociation and recombination rate constants, respectively, Equation (2)
$k_{d}$ and $k_{r}$	Dissociation and recombination rate constants for water, respectively
L	Mass transfer coefficient
$l_{hf}$	Effective length of the hollow-fiber membrane
m	Membrane mass
$N_{hf}$	Number of hollow-fiber membranes in the dialyzer
P	Membrane diffusion permeability
R	Gas constant
$S_{mb}$	Effective surface area of the hollow-fiber membrane in the dialyzer
T	Absolute temperature
t	Time
$V^{A}$ and $V^{B}$	Volume of the diluate and dialysate compartments, respectively
W	Mass fraction of water in the membrane
WC	Membrane water content
x	Coordinate normal to the membrane surface
z	Charge
Greek Symbols	
α	Structural parameter characterizing the arrangement of pores relative to the transport axis
δ	Diffusion boundary layer thickness
ρ	Density of the dry membrane
φ	Electric potential
Superscripts/subscripts	
*A*	Diluate compartment
*B*	Dialysate compartment
*dry*	Dry state
*sw*	Swollen state
